# Impact of self-reported physical activity and health promotion behaviors on lung cancer survivorship

**DOI:** 10.1186/s12955-016-0461-3

**Published:** 2016-04-29

**Authors:** Jeff A. Sloan, Andrea L. Cheville, Heshan Liu, Paul J. Novotny, Jason A. Wampfler, Yolanda I. Garces, Matthew M. Clark, Ping Yang

**Affiliations:** Department of Health Sciences Research, 200 First St SW, Rochester, MN 55905 USA; Department of Physical Medicine and Rehabilitation, Rochester, USA; Department of Radiation Oncology, Rochester, USA; Department of Psychiatry and Psychology, Mayo Clinic, Rochester, MN 55905 USA

## Abstract

**Background:**

There is some initial evidence that an enhanced physical activity level can improve fquality of life, and possibly survival among patients with lung cancer. The primary aim of this project was to evaluate the impact of physical activity on the quality and quantity of life of lung cancer survivors.

**Methods:**

Between January 1, 1997, and December 31, 2009, a total of 1466 lung cancer survivors completed a questionnaire with patient-reported outcomes for quality of life (QOL), demographics, disease and clinical characteristics, and a measure of physical activity (Baecke Questionnaire). Chi-square tests compared lung cancer survivors who reported being physically active versus not on a variety of the other covariates. Kaplan-Meier estimates and Cox models evaluated the prognostic importance of physical activity level on Overall Survival (OS).

**Results:**

Roughly half of the lung cancer survivors had advanced stage disease at the time of survey. Treatment prevalence rates were 61, 54, and 33 % for surgery, chemotherapy and radiotherapy, respectively. The majority (77 %) of survivors reported themselves as physically active. Physically active survivors reported greater activity across all individual Baecke items. Lung cancer survivor-reported QOL indicated the benefits of physical activity in all domains. Survivors receiving chemotherapy or radiation at the time of questionnaire completion were less likely to be physically active (74 and 73 % respectively). In contrast, 84 % of surgical patients were physically active. Disease recurrence rates were the same for physically active and inactive patients (81 % vs 82 %, *p* = 0.62). Physically active patients survived an average of 4 more years than those who were not physically active (8.4 years versus 4.4 years respectively, log rank *p* < 0.0001).

**Conclusions:**

Being physically active was related to profound advantages in QOL and survival in a large sample of lung cancer survivors.

## Background

There is some initial evidence that an enhanced physical activity level can improve feelings of well-being, quality of life, and possibly survival among patients with lung cancer [1]. In general the impact of exercise behaviors following a cancer diagnoses has been most extensively investigated for patients with breast and gastrointestinal malignancies [2, 3]. However, accruing evidence suggests that exercise behaviors may be an important and actionable determinant of lung cancer outcomes. The potential for a novel, non-toxic therapy warrants attention, since lung cancer, the most common cause of cancer death, has a tendency for late-stage diagnosis and, despite novel drug therapies, rapid, morbid, and inexorable progression [4].

To date a range of both cross-sectional and longitudinal studies have examined associations between aerobic fitness and important outcomes at seminal points along the lung cancer trajectory. With few exceptions, investigators have characterized aerobic fitness at a single time point using maximal oxidative capacity (V0_2max_) or 6 min walk distance (6MWD). Cross sectional investigations have shown that aerobic fitness is associated with post-operative symptom burden and quality of life among patients with operable lung cancer [5, 6]. Longitudinal investigations have demonstrated that aerobic fitness predicts peri-operative complication rates for lung cancer surgeries, [7–9] as well as overall survival for both operable [1] and inoperable [10] lung cancer. Encouragingly, pilot studies have found exercise-based pulmonary rehabilitation programs, both pre- and post-operative, to be well tolerated and to enhance aerobic fitness [9, 11–13]. Even patients with late stage lung cancer derive benefit from exercise training as manifest in improved a functional capacity and a reduced symptom burden [14–16].

Despite mounting evidence attesting the benefits of enhanced physical activity among lung cancer survivors, the longitudinal impact of shifting activity levels following a lung cancer diagnosis has yet to be examined using repeated measures. Our group previously reported that endorsement of regular physical activity among 272 long-term lung cancer survivors was associated with higher scores in overall QOL and all QOL sub-domains, as well as a reduced symptom burden [26]. The primary aim of this project was to prospectively examine the relationship of physical activity level with quality of life and overall survival across multiple time points in a larger cohort of 1466 long-term lung cancer survivors. Secondary aims included examining the influence of treatment and demographic variables on these relationships and describing differences between survivors who did and did not characterize themselves as being physically active.

## Methods

The Mayo Clinic Epidemiology and Genetics of Lung Cancer Research Program has enrolled and prospectively followed patients either diagnosed with and/or treated for lung cancer at Mayo Clinic, Rochester, Minnesota since its inception in 1997. Between January 1, 1997, and December 31, 2009, over 10,000 patients have been enrolled. Procedures for identifying and following lung cancer patients enrolled in this program have been previously described [17]. Patient follow-up was accomplished by mailed questionnaire beginning at six months after diagnosis and annually thereafter.

Quality of life was assessed at all follow-up time points by means of one item from the Lung Cancer Symptom Scale (LCSS) [18, 19]. The overall QOL item served as the primary endpoint in the current study. In the primary analysis overall QOL is considered as a continuous variable, taking integer values from 0 to 100. Previously, The North Central Cancer treatment Group (NCCTG) Lung Cancer Committee compared alternative QOL assessments (the European Organization for the Research and Treatment of Cancer Quality of Life Questionnaire and Lung Cancer Module(EORTC-QLQ-LC13), the Functional Assessment of Cancer Therapy - Lung (FACT-L), and the Lung Cancer Symptom Scale(LCSS)) using a series of phase II clinical trials (NCCTG trials 95-20-53, 95-24-52, 96-24-51, 98-24-52). Ultimately it was found that the LCSS performed as well as the other two assessments and used fewer items to obtain the same information [20]. A pooled analysis of lung cancer studies indicated that the single-item QOL assessments developed within the NCCTG were more sensitive to change than longer, multi-item assessments [21]. This study also demonstrated that the relationship between QOL assessments and toxicity was modest at best and that clinically meaningful changes in QOL preceded adverse events captured by the Common Toxicity Criteria(CTC) by two to three months.

These single-item assessments have become the most-used assessment in all NCI-sponsored cancer control studies [22] and have been validated extensively against more involved assessment processes [23, 24]. Normative data have been obtained from various clinical populations enrolled in NCCTG clinical trials and from healthy participants attending an NCCTG annual meeting. In assessing overall QOL on a 0–100 point scale, healthy volunteers will average about 82, hospice patients will average 78, advanced cancer patients will average somewhere between 60 and 75, newly diagnosed patients will average between 50 and 60. A score of 50 or below is indicative of a need for immediate exploration and intervention for the QOL deficit [25]. This cutoff has been validated both by our research team [21, 26] and independently by others [27–29]. We recently published data indicating that these measures held prognostic power specifically among lung cancer patients [30]. The NCCTG has included Linear Analog Scale Assessment(LASA) measures for overall QOL and fatigue in all phase II and phase III clinical trials since 2009 as an independent prognostic factor independent of performance status.

Self-reported physical activity and health promotion behaviors were provided by the lung cancer survivors using the modified Baecke questionnaire for physical activity [31]. First developed for epidemiological studies of physical activity in a Dutch population [32], the 16-item questionnaire consists of three subscales: 1) physical activity at work; 2) sport during leisure time; and 3) physical activity during leisure time excluding sport [33]. It has been used in healthy, elderly, and chronic fatigue populations successfully [34–36]. Recent studies have idenitifed issues with its validity and reliability, but it remains the most often-used questionnaire for assessing physical activity in epidemiological studies [37]. Specifically, it has been suggested that the Baecke questionnaire is best used to differentiate between active and inactive individuals rather than to differentiate active people into low and high activity level categories [38].

The study sample included 1466 lung cancer survivors who completed the Baecke questionnaire at least once. The study sample used the first Baecke data recorded by each survivor to define the participant’s physical activity level. We found little change over time in the Baecke scores (data not shown). By using the first observed Baecke scores, we acknowledge that the time since diagnosis and years studied will be variable. The scoring algorithm for the Baecke questionnaire was untenable because of a large amount of non-interpretable data. Our study team went through an extensive data cleaning operation wherein we made operational definitions and decisions as to how to record various eccentricities in the data. For example, patients provided multiple sports when asked for their primary sport activity, reported gambling as a sport, or provided Illogical combinations such as indicating they engaged in physical exercise during leisure time regularly for a total of zero minutes per day. As a result it became clear that we would only be able to present some subscale data and in reality could only analyze individual questions with confidence. The study protocol was reviewed and approved by the Mayo Clinic Institutional Review Board. Ethics approval of this study was granted by Mayo Clinic Institutional Review Board. Written informed consent was obtained from all participants.

Power considerations with 1466 lung cancer survivors, any percentage reported on the entire sample is accurate to within 2.6 with 95 % confidence. Any mean reported on such a sample is accurate to within 5 % times the standard deviation(SD) of the continuous variable’s distribution, which is classified as a small effect size [39]. For example, it is known that our overall QOL scores that range from 0–100 have a standard deviation of roughly 16.7 points. This would mean that the mean QOL reported for this sample will be accurate to within 1 point on the 0–100 point scale.

Covariates considered in this study can be broadly grouped into demographic (age, gender, race, comorbidities), social (employment status, marital status, years of education), smoking history (pack years, never, former, recent quitter, still smoking) disease-related (histology, stage, grade), and treatment-related (chemotherapy, radiation, surgery) characteristics. Smoking classification was assessed in several ways. First was pack years, defined as the number of packs of cigarettes smoked over time. For example, a participant who smoked one pack of cigarettes per day for 20 years, would have a 20 year pack history. Participants were also classified according to smoking status at the time they completed the survey packet: never smoker (less than 100 life time cigarettes), former smoker (quit more than 12 months), recent quitter (quit more than 30 days but less than 12 months), or current smoker (any tobacco usage in the past 30 days).

Model Building included Cox proportional hazards models (forward and backward stepping, saturated, and stepwise approaches) for relating exercise to survival while controlling for the covariates listed above. Cluster analysis was employed to identify correlated symptoms and treatment/disease status, thus reduce dimensionality of the numerous covariate influences that arose from the survival models.

## Results

Demographics and clinical data are summarized in Table 1 for the 1466 lung cancer survivors comprising our sample. Survivors ranged in age from 18 to 93 years of age with an average age of 66 years (SD = 11 years). The sample was equally divided between the genders and 93 % of the sample was Caucasian. Over 90 % graduated high school and the majority was married (78 %). Follow-up ranged as long as 9 years but averaged 3 years, and 35 % of the possible study participants were dead at the time of study follow-up. Smoking behavior past or present was prevalent (83 %) although 17 % of the lung cancer survivors reported that they had never smoked. For those that smoked the average pack years was 47 years. Roughly half of the survivors had advanced stage disease at the time of observation. Treatment prevalence rates were 61, 54, and 33 % for surgery, chemotherapy and radiotherapy respectively.

**Table 1:** Participant characteristics for 1466 lung cancer survivors by Baecke physical activity data

Participant characteristic	Non-physically active (*N* = 331)	Physically active (*N* = 1135)	Total (*N* = 1466)
Age at diagnosis			
*N*	331	1135	1466
Mean (SD)	68.7 (9.73)	64.9 (10.82)	65.7 (10.7)
Range	(35.0–91.0)	(18.0–93.0)	(18–93)
Gender			
Female	158 (47.7 %)	568 (50 %)	726 (49.5 %)
Male	173 (52.3 %)	567 (50 %)	740 (50.5 %)
Race			
Caucasian	311 (94 %)	1059 (93.3 %)	1370 (93.5 %)
Hispanic	4 (1.2 %)	8 (0.7 %)	12 (0.8 %)
Alaskan/Indian	15 (4.5 %)	54 (4.8 %)	69 (4.7 %)
Black	1 (0.3 %)	3 (0.3 %)	4 (0.3 %)
Asian/Pacific Islander	0 (0.0 %)	6 (0.5 %)	6 (0.4 %)
Unknown	0 (0.0 %)	5 (0.4 %)	5 (0.3 %)
Years of schooling completed			
N	67	307	374
Mean (SD)	13.0 (2.35)	13.6 (2.41)	13.5 (2.4)
Range	(6.0–17.0)	(5.0–18.0)	(5.0–18.0)
Marital status			
Missing	62	185	247
Single	9 (3.3 %)	47 (4.9 %)	56 (4.6 %)
Married	194 (72.1 %)	751 (79.1 %)	945 (77.5 %)
Divorced	27 (10 %)	75 (7.9 %)	102 (8.4 %)
Widowed	39 (14.5 %)	75 (7.9 %)	114 (9.4 %)
Life partner	0 (0.0 %)	2 (0.2 %)	2 (0.2 %)
Vital status (as of 2/2010)			
Alive	167 (50.5 %)	787 (69.3 %)	954 (65.1 %)
Dead	164 (49.5 %)	348 (30.7 %)	512 (34.9 %)
Time to last FU (as of 2/2010) in years			
N	331	1135	1466
Mean (SD)	2.7 (2.15)	3.3 (2.43)	3.1 (2.38)
Range	(0.1–8.6)	(0.0–9.0)	2.2
Cigarette smoking status			
Never	36 (10.9 %)	218 (19.2 %)	254 (17.3 %)
Former smoker	165 (49.8 %)	615 (54.2 %)	780 (53.2 %)
Current smoker	129 (39 %)	294 (25.9 %)	423 (28.9 %)
Some smoking history	1 (0.3 %)	8 (0.7 %)	9 (0.6 %)
Pack-Years			
N	292	910	1202
Mean (SD)	53.2 (34.44)	45.4 (28.29)	47.3 (30.07)
Range	(0.5–208.0)	(0.0–180.0)	(0.0–208.0)
Condensed grade			
Missing	1	6	7
1 = Well differentiated	58 (17.6 %)	328 (29.1 %)	386 (26.5 %)
2 = Moderately differentiated	104 (31.5 %)	414 (36.7 %)	518 (35.5 %)
3 = Poorly differentiated	142 (43 %)	321 (28.4 %)	463 (31.7 %)
4 = Non-gradeable	26 (7.9 %)	66 (5.8 %)	92 (6.3 %)
Stage			
Missing	6	5	11
Limited	27 (8.3 %)	36 (3.2 %)	63 (4.3 %)
Extensive	22 (6.8 %)	30 (2.7 %)	52 (3.6 %)
Stage IA	49 (15.1 %)	320 (28.3 %)	369 (25.4 %)
Stage IB	48 (14.8 %)	186 (16.5 %)	234 (16.1 %)
Stage IIA	6 (1.8 %)	24 (2.1 %)	30 (2.1 %)
Stage IIB	17 (5.2 %)	68 (6 %)	85 (5.8 %)
Stage IIIA	42 (12.9 %)	124 (11 %)	166 (11.4 %)
Stage IIIB	45 (13.8 %)	116 (10.3 %)	161 (11.1 %)
Stage IV	69 (21.2 %)	226 (20 %)	295 (20.3 %)
T (tumor) of TNM staging			
Missing	69	176	245
No primary tumor	6(2.3 %)	10(1.0 %)	16(1.3 %)
Tumor < = 3 cm	83 (31.7 %)	420 (43.8 %)	503 (41.2 %)
Tumor > 3 cm	92 (35.1 %)	309 (32.2 %)	401 (32.8 %)
Invades chest wall	14 (5.3 %)	54 (5.6 %)	68 (5.6 %)
Invades mediastinum	56 (21.4 %)	137 (14.3 %)	193 (15.8 %)
Cannot be assessed	11 (4.2 %)	29 (3 %)	40 (3.3 %)
N (nodes) of TNM staging			
Missing	51	123	174
No nodal mets	122 (43.6 %)	611 (60.4 %)	733 (56.7 %)
In Peribr/Hilar	30 (10.7 %)	98 (9.7 %)	128 (9.9 %)
In Medias/Subcarinal	97 (34.6 %)	210 (20.8 %)	307 (23.8 %)
Mets in contralaterl	22 (7.9 %)	76 (7.5 %)	98 (7.6 %)
Nodes Unassessable	9 (3.2 %)	17 (1.7 %)	26 (2 %)
Surgery			
Missing	12	27	39
No	179 (56.1 %)	381 (34.4 %)	560 (39.2 %)
Yes	140 (43.9 %)	727 (65.6 %)	867 (60.8 %)
Surgery within 6 months			
Missing	12	27	39
No	189 (59.2 %)	435 (39.3 %)	624 (43.7 %)
Yes	130 (40.8 %)	673 (60.7 %)	803 (56.3 %)
Chemotherapy			
Missing	12	27	39
No	120 (37.6 %)	542 (48.9 %)	662 (46.4 %)
Yes	199 (62.4 %)	566 (51.1 %)	765 (53.6 %)
Chemotherapy within 6 months			
Missing	12	27	39
No	166 (52 %)	686 (61.9 %)	852 (59.7 %)
Yes	153 (48 %)	422 (38.1 %)	575 (40.3 %)
Radiation			
Missing	12	27	39
No	193 (60.5 %)	769 (69.4 %)	962 (67.4 %)
Yes	126 (39.5 %)	339 (30.6 %)	465 (32.6 %)
Radiation within 6 months			
Missing	12	27	39
No	230 (72.1 %)	875 (79 %)	1105 (77.4 %)
Yes	89 (27.9 %)	233 (21 %)	322 (22.6 %)

Physical activity level of survivors was dichotomized as physically active or not physically active by the readiness to change scale as described above. The majority (77 %) of the 1466 patients who provided a physical activity classification (6 out of the original 1472 patients did not) reported themselves as being in the action stage for physical activity based on this classification. The individual item results for the Baecke activity questionnaire are given in Table 2. Survivors who were physically active reported significantly different results for almost all of the individual Baecke items. Almost 62 % of the patients were retired, while 28 % identified themselves as being employed. Roughly 45 % of patients reported they participated regularly in sports or exercise an average of 6 h per week. Roughly half of the survivors indicated that they thought they participated in sport activity less than others. Over three quarters of the survivors reported that they engaged in cycling on at least a semi-regular basis. Roughly 15 % (208 out 1427) of patients changed their level of physical activity over time.

**Table 2:** Individual Baecke item overall distributions by physical activity level

	Non-physically active (*N* = 331)	Physically active (*N* = 1135)	Total (*N* = 1466)	*p* value
Retired				<0.0001^*^
Missing	68	153	221	
No	56 (21.3 %)	421 (42.9 %)	477 (38.3 %)	
Yes	207 (78.7 %)	561 (57.1 %)	768 (61.7 %)	
Employed				<0.0001^*^
Missing	4	6	10	
No	285 (87.2 %)	757 (67.1 %)	1042 (71.6 %)	
Yes	42 (12.8 %)	372 (32.9 %)	414 (28.4 %)	
At work - sit				0.0003^*^
Missing	283	742	1025	
Never	1 (2.1 %)	24 (6.1 %)	25 (5.7 %)	
Seldom	0 (0 %)	57 (14.5 %)	57 (12.9 %)	
Sometimes	7 (14.6 %)	105 (26.7 %)	112 (25.4 %)	
Often	27 (56.3 %)	129 (32.8 %)	156 (35.4 %)	
Always/Very Often	13 (27.1 %)	78 (19.8 %)	91 (20.6 %)	
At work - stand				
Missing	289	745	1034	
Never	14 (33.3 %)	39 (10.0 %)	53 (12.3 %)	
Seldom	28 (66.7 %)	126 (32.3 %)	154 (35.6 %)	
Sometimes	0 (0 %)	140 (35.9 %)	140 (32.4 %)	
Often	0 (0 %)	85 (21.8 %)	85 (19.7 %)	
At work - walk				<0.0001^*^
Missing	286	745	1031	
Never	15 (33.3 %)	35 (9.0 %)	50 (11.5 %)	
Seldom	30 (66.7 %)	117 (30.0 %)	147 (33.8 %)	
Sometimes	0 (0 %)	166 (42.6 %)	166 (38.2 %)	
Often	0 (0 %)	72 (18.5 %)	72 (16.6 %)	
At work - lift				0.2175^*^
Missing	314	860	1174	
Never	12 (66.7 %)	138 (50.2 %)	150 (51.4 %)	
Seldom	5 (33.3 %)	81 (29.5 %)	86 (29.5 %)	
Sometimes	0 (0 %)	42 (15.3 %)	42 (14.4 %)	
Often	0 (0 %)	14 (5.1 %)	14 (4.8 %)	
At work - sweat				0.0160^*^
Missing	307	851	1158	
Never	14 (58.3 %)	113 (39.8 %)	127 (41.2 %)	
Seldom	10 (41.7 %)	95 (33.5 %)	105 (34.1 %)	
Sometimes	0 (0 %)	60 (21.1 %)	60 (19.5 %)	
Often	0 (0 %)	16 (5.6 %)	16 (5.2 %)	
After work - tired				0.0694^*^
Missing	284	742	1026	
Never	3 (6.4 %)	37 (9.4 %)	40 (9.1 %)	
Seldom	12 (25.5 %)	155 (39.4 %)	167 (38.0 %)	
Sometimes	15 (31.9 %)	122 (31.0 %)	137 (31.1 %)	
Often	17 (36.2 %)	79 (20.1 %)	96 (21.8 %)	
Work compared to others				0.0009^*^
Missing	285	734	1019	
Much heavier	1 (2.2 %)	26 (6.5 %)	27 (6.0 %)	
Heavier	3 (6.5 %)	50 (12.5 %)	53 (11.9 %)	
As heavy	12 (26.1 %)	155 (38.7 %)	167 (37.4 %)	
Lighter	19 (41.3 %)	131 (32.7 %)	150 (33.6 %)	
Much lighter	11 (23.9 %)	39 (9.7 %)	50 (11.2 %)	
Sports or exercise				<0.0001^*^
Missing	15	20	35	
No	316 (100 %)	459 (41.2 %)	775 (54.2 %)	
Yes	0 (0 %)	656 (58.8 %)	656 (45.8 %)	
Second sport or exercise				<0.0001^*^
Missing	275	479	754	
No	56 (100 %)	436 (66.5 %)	492 (69.1 %)	
Yes	0 (0 %)	220 (33.5 %)	220 (30.9 %)	
Sport activity compared to others				<0.0001^*^
Missing	63	84	147	
Much more	0 (0 %)	72 (6.9 %)	72 (5.5 %)	
More	0 (0 %)	221 (21.0 %)	221 (16.8 %)	
The Same	36 (13.4 %)	345 (32.8 %)	381 (28.9 %)	
Less	97 (36.2 %)	272 (25.9 %)	369 (28.0 %)	
Much Less	135 (50.4 %)	141 (13.4 %)	276 (20.9 %)	
Comparison based on frequency				0.0057^*^
Missing	202	511	713	
No	8 (6.2 %)	10 (1.6 %)	18 (2.4 %)	
Yes	121 (93.8 %)	614 (98.4 %)	735 (97.6 %)	
Comparison based on intensity				0.2262^*^
Missing	242	740	982	
No	11 (12.4 %)	33 (8.4 %)	44 (9.1 %)	
Yes	78 (87.6 %)	362 (91.6 %)	440 (90.9 %)	
Comparison based on duration				0.5282^*^
Missing	231	746	977	
No	9 (9.0 %)	28 (7.2 %)	37 (7.6 %)	
Yes	91 (91.0 %)	361 (92.8 %)	452 (92.4 %)	
Leisure - sweat				<0.0001^*^
Missing	20	55	75	
Very often	0 (0 %)	55 (5.1 %)	55 (4.0 %)	
Often	0 (0 %)	133 (12.3 %)	133 (9.6 %)	
Sometimes	94 (30.2 %)	345 (31.9 %)	439 (31.6 %)	
Seldom	119 (38.3 %)	364 (33.7 %)	483 (34.7 %)	
Never	98 (31.5 %)	183 (16.9 %)	281 (20.2 %)	
Leisure - Yard or housework				<0.0001^*^
Missing	17	17	34	
Very often	0 (0 %)	355 (31.8 %)	355 (24.8 %)	
Often	0 (0 %)	433 (38.7 %)	433 (30.2 %)	
Sometimes	176 (56.1 %)	211 (18.9 %)	387 (27.0 %)	
Seldom	60 (19.1 %)	77 (6.9 %)	137 (9.6 %)	
Never	78 (24.8 %)	42 (3.8 %)	120 (8.4 %)	
Leisure - watch TV				<0.0001^*^
Missing	9	23	32	
Very often	118 (36.6 %)	206 (18.5 %)	324 (22.6 %)	
Often	96 (29.8 %)	415 (37.3 %)	511 (35.6 %)	
Sometimes	82 (25.5 %)	381 (34.3 %)	463 (32.3 %)	
Seldom	13 (4.0 %)	97 (8.7 %)	110 (7.7 %)	
Never	13 (4.0 %)	13 (1.2 %)	26 (1.8 %)	
Leisure - walk				<0.0001^*^
Missing	17	22	39	
Very often	9 (2.9 %)	166 (14.9 %)	175 (12.3 %)	
Often	28 (8.9 %)	355 (31.9 %)	383 (26.8 %)	
Sometimes	122 (38.9 %)	390 (35.0 %)	512 (35.9 %)	
Seldom	106 (33.8 %)	166 (14.9 %)	272 (19.1 %)	
Never	49 (15.6 %)	36 (3.2 %)	85 (6.0 %)	
Leisure - bicycle				<0.0001^*^
Missing	27	66	93	
Very often	1 (0.3 %)	24 (2.2 %)	25 (1.8 %)	
Often	4 (1.3 %)	55 (5.1 %)	59 (4.3 %)	
Sometimes	14 (4.6 %)	148 (13.8 %)	162 (11.8 %)	
Seldom	37 (12.2 %)	186 (17.4 %)	223 (16.2 %)	
Never	248 (81.6 %)	656 (61.4 %)	904 (65.8 %)	
Leisure - Walk/bike minutes per day				<0.0001^**^
N	171	695	866	
Mean (SD)	6.9 (15.1)	18.7 (32.7)	16.3 (30.4)	
Median	0.0	10.0	5.0	
Q1, Q3	0.0, 10.0	0.0, 30.0	0.0, 30.0	
Range	(0–120)	(0–600)	(0–600)	

^*^Chi-square test, ^**^Wilcoxon

Co-morbidities and cancer stage impact on physical activity indicated that patients with co-morbidities were more likely to not be physically active versus those who did not have co-morbidities (23 % versus 18 %, *p* = 0.14 respectively). Similarly, patients with early stage disease were more likely to be physically active than those with late stage disease (83 % versus 72 %, *p* < .001 respectively).

Lung cancer treatment impact on physical activity is provided in Table 3. Participants who were physically active were more likely to be surgical patients than receiving chemotherapy or radiation (43, 11 and 11 %, respectively). Similarly, more of the surgical patients (87 %) were physically active than for the other treatments (73 %) For survivors who had multiple treatments, 14 % of patients changed in the degree of physical activity level after 1 year.

**Table 3:** Treatment type by physical activity level

	Non-physically active (*N* = 331)	Physically active (*N* = 1135)	Total (*N* = 1466)	*p* value
Treatment				<0.0001^*^
Missing	12	27	39	
Surgery alone	90 (6.3 %)	490 (34.3 %)	580(40.6 %)	
Chemo or radio alone	75 (5.3 %)	155 (10.9 %)	230 (16.1 %)	
Surgery and chemo or radio	29 (2.0 %)	121 (8.5 %)	150(10.5 %)	
Chemo plus radio	67 (4.7 %)	161 (11.3 %)	228 (16.0 %)	
Other	58 (4.1 %)	181 (12.7 %)	239 (16.8 %)	

^*^Chi-square test

Lung cancer survivor-reported QOL indicated the benefits of physical activity in all domains (Table 4). Overall QOL and physical QOL differed by more than 15 points on a 0–100 point scale, roughly equivalent to one standard deviation, a huge effect size. Emotional and mental well-being differed by roughly 9 points, larger than a clinically meaningful effect size of 50 % times the standard deviation. Lung cancer survivors who were physically active also reported less pain frequency, pain severity, dry coughing, coughing with phlegm, SOB, and fatigue.

**Table 4:** Quality of life by physical activity level

	Non-physically active (*N* = 331)	Physically active (*N* = 1135)	Total (*N* = 1466)	*p* value^*^
Emotional well being				<0.0001
*N*	299	1015	1314	
Mean (SD)	69.3 (22.1)	77.8 (19.4)	75.9 (20.3)	
Deficiency (<=50)	92 (30.8 %)	150 (14.8)		
Physical well being				<0.0001
*N*	299	1015	1314	
Mean (SD)	54.2 (22.9)	70.8 (20.2)	67.1 (21.9)	
Deficiency (<=50)	166 (55.5 %)	229 (22.6 %)		
Mental well being				<0.0001
*N*	299	1016	1315	
Mean (SD)	71.7 (21.70)	80.1 (18.5)	78.2 (19.6)	
Deficiency (<=50)	82 (27.4 %)	120 (11.8 %)		
Overall QOL				<0.0001
*N*	299	1014	1313	
Mean (SD)	59.3 (23.3)	75.6 (18.8)	71.9 (21.1)	
Deficiency (<=50)	140 (46.2 %)	161 (16.0 %)		
Frequency of pain				<0.0001
*N*	296	1013	1309	
Mean (SD)	57.0 (30.5)	67.4 (29.1)	65.0 (29.7)	
Deficiency (<=50)	145 (49.0 %)	339 (33.5 %)		
Severity of pain				<0.0001
*N*	296	1014	1310	
Mean (SD)	61.4 (27.1)	72.7 (24.7)	70.2 (25.7)	
Deficiency (<=50)	129 (43.6 %)	258 (25.4 %)		
Frequency of dry coughing				0.0035
*N*	299	1016	1315	
Mean (SD)	67.0 (27.3)	71.7 (27.2)	70.6 (27.3)	
Deficiency (<=50)	96 (32.1 %)	273 (26.9 %)		
Frequency of coughing with phlegm				<0.0001
*N*	297	1011	1308	
Mean (SD)	67.3 (29.4)	75.6 (26.8)	73.7 (27.6)	
Deficiency (<=50)	102 (34.3 %)	228 (22.5 %)		
Shortness of breath				<0.0001
*N*	299	1018	1317	
Mean (SD)	46.9 (27.7)	59.5 (27.4)	56.6 (28.0)	
Deficiency (<=50)	191 (63.9 %)	448 (44.0 %)		
Level of fatigue				<0.0001
*N*	299	1018	1317	
Mean (SD)	39.6 (23.1)	53.9 (24.3)	50.7 (24.8)	
Deficiency (<=50)	227 (75.9 %)	555 (54.5 %)		

^*^Wilcoxon

Physical activity by time until death analysis indicated that patients who were within 6 months of death less active than patients who were 6 months to a year, versus more than a year away from death (72, 76, 78 % respectively and *p* = 0.80).

**Fig. 1:**
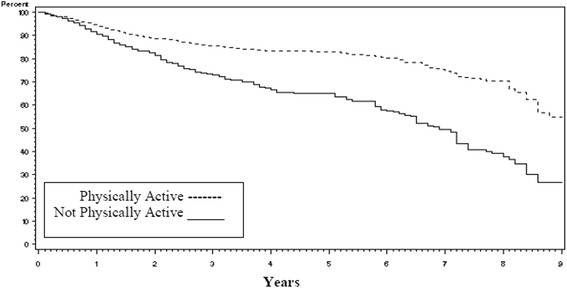
Kaplan-Meier survival curves for lung cancer survivors by level of physical activity

**Fig. 2:**
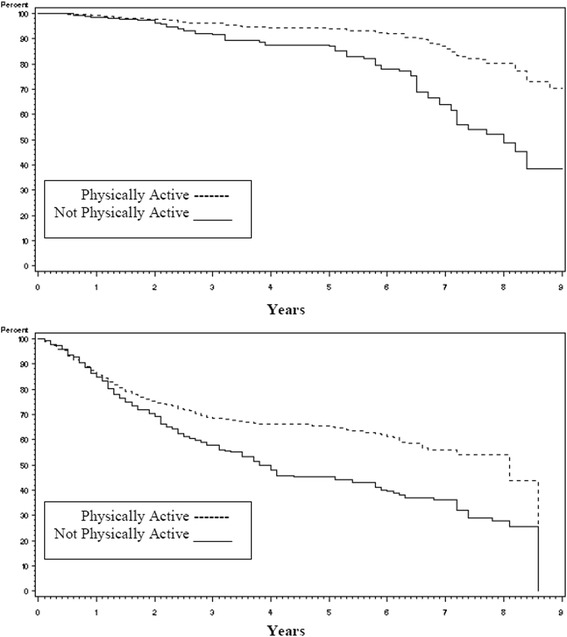
Kaplan-Meier survival curves for lung cancer survivors by stage of disease (early vs late)

**Fig. 3:**
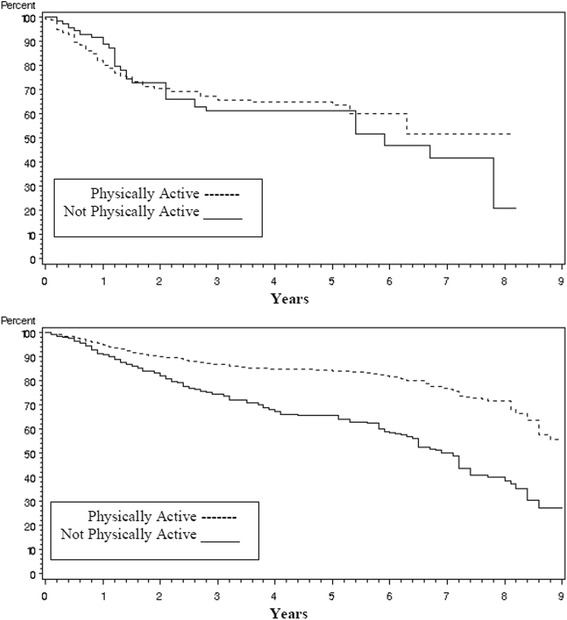
Kaplan-Meier survival curves for lung cancer survivors by type of lung cancer (Non-Small Cell Lung Cancer(NSCLC) vs Small Cell Lung Cancer(SCLC))

Disease recurrence rates were the same whether the patients reported being physically active (81 %) or not physically active (82 %) (Chi-square test *p* = 0.62). Overall survival however was profoundly different as seen in Fig. 1 with those who were physically active surviving an average of 4 more years than those who were not physically active (8.4 years versus 4.4 years respectively, logrank *p* < 0.0001). The impact of being physically active on survival was consistent across disease stage (early versus late, Fig. 2) and type of lung cancer (Non-Small Cell Lung Cancer (NSCLC) vs. Small Cell Lung Cancer (SCLC), Fig. 3). When covariates (age at diagnosis, gender, race, disease stage, treatment, smoking status, and pack years) were added to the survival analysis via a Cox regression model, the hazard ratio for non-physically active patients to physically active patients was 1.29 (Table 5, 95 % CI =1.00 to 1.67, *p* = 0.05).

**Table 5:** Cox regression model

Parameter	Hazard ratio	95 % hazard ratio confidence limits		*P* value
Non physically active	1.294	1.003	1.669	0.0472
Age at diagnosis	1.019	1.007	1.031	0.0021
Female	0.828	0.662	1.035	0.0976
Surgery alone	0.414	0.283	0.606	<.0001
Chemo or radio alone	2.333	1.665	3.268	<.0001
Surgery and chemo or radio	0.728	0.468	1.134	0.1602
Chemo plus radio	1.463	1.027	2.085	0.0353
Never smoker	0.890	0.620	1.279	0.5292
Former smoker	1.062	0.824	1.369	0.6418
Overall QOL	0.992	0.987	0.998	0.0057
Fatigue	0.992	0.985	0.998	0.0094
Coughing	1.002	0.997	1.007	0.4913
Shortness of breath	1.003	0.998	1.008	0.2170
Pain	0.997	0.992	1.001	0.1594
Stage I	0.329	0.226	0.479	<.0001
Stage II	0.405	0.250	0.656	0.0002
Stage III/Limited	0.551	0.419	0.726	<.0001

**Fig. 4:**
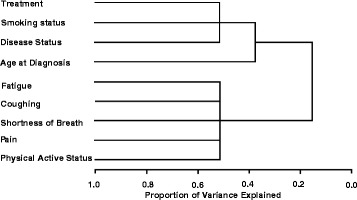
Cluster dendogram for lung cancer survivors

Cluster analysis of the nine potentially important variables (Fig. 4) indicated that two clusters formed readily related to symptoms (pain, fatigue, cough, shortness of breath, physical active status) and to treatment/disease status (treatment type, disease stage, smoking status). Cluster analysis/logistic regression indicated that the first characteristic differentiating active patients from inactive patients was fatigue, followed by pain, treatment, age at diagnosis and smoking status. Collectively these five items accounted for 19 % of the variance in physical activity.

## Discussion

This large prospective epidemiological study of lung cancer survivors provides further support for the premise that being physical active is beneficial to both quality and quantity of life. Virtually all QOL domains studied indicated clinically meaningful advantages for lung cancer survivors reporting being physically active versus those who were not physically active.

These findings are consistent with previous literature in that the benefits of being active for lung cancer survivors are similar to those for the general population. The differences were profound in terms of the effect size benefit for those who are physically active. Recent studies of the most prevalent and concerning symptoms among cancer patients indicate that similar to our patients, fatigue, pain and insomnia are major impacts on the QOL of lung cancer patient survivors. Smoking continues to be a profound indicator of well-being for cancer patients. The results are also consistent with our previous work on the prognostic power of overall QOL on the survival of lung cancer patients30 in that it highlights a natural linkage between physical activity, health promotion behavior, and QOL which together in turn contribute to the quality and quantity of survival in patients with lung cancer.

This report provides new information regarding the specific issues facing lung cancer survivors. Encouragingly, the majority of lung cancer survivors do remain physically active. The deficits observed however are specific to lung cancer and different from those expressed by breast cancer survivors for example. Further, this study identified that physical activity in general was related to the level of symptoms experienced and the type of treatments received. In particular, surgical patients can be expected to be more likely to be physically active because they are likely to be earlier stage and less ill than other patients.

This study has limitations in that it is an observational rather than a controlled experimental layout. There could be concomitant influences alongside the physical activity reporting that could account for the majority of the apparent impact. For example, performance status and stage of disease could play a role in determining if a person is physically active or not. This is unlikely the case in this study however, because even once these covariates had been included in the survival model, the significance of the physical activity level remained.

Further, it is acknowledged that obtaining accurate measures of physical activity is challenging. In this study we encountered a major barrier in the quality of the data returned for the Baecke questionnaire. Presumably in previous validation studies, participants were given the questionnaire in the presence of study assistants who could help them answer the relatively complex and involved questions of the Baecke questionnaire. Nonetheless, we were able to analyze the individual items and saw that the results were amazingly consistent for all 16 questions. Future research is needed to make this challenging task easier for patients and scientists to obtain accurate and consistent estimates of physical activity, especially since the impact of physical activity has demonstrated to have potentially profound impact on patient well-being.

## Conclusion

Some recent work described the ability to integrate objective measures in large cohort design, the future studies should employ objective measures of physical activity, such as the accelerometry [40, 41].

## Abbreviations

LCSS: Lung Cancer Symptom Scale
NCCTG: North Central Cancer treatment Group
QOL: quality of life
